# The complete chloroplast genome sequences of *Dolichandrone spathacea* (L. F.) K. Schum., a semi-mangrove plant

**DOI:** 10.1080/23802359.2021.1901619

**Published:** 2021-03-26

**Authors:** Ting Yu, Si-Yu Ren, Xin-Rui Wang, Li Xu, Jiu-Heng Xu, Gong-Tao Ding, Li-Ming Tang, Dong-Xu Zhang, Wen-Bin Guan

**Affiliations:** a School of Ecology and Nature Conservation, Beijing Forestry University, Beijing, PR China; b Forestry Department of Guangxi, Nanning, PR China;; c Protected Agricultural Technology Development Center, Shanxi Datong University, Shanxi, PR China

**Keywords:** *Dolichandrone spathacea*, Bignoniaceae, chloroplast genome, phylogenetic analysis, semi-mangrove plant

## Abstract

*Dolichandrone spathacea*(L. F.) K. Schum. is an excellent tree species for coastal protection forests. In this study, the complete chloroplast genome sequence of *D. spathacea* was obtained through high-throughput sequencing. The length of chloroplast genome was 159,156 bp in length, containing a large single-copy region (LSC) of 86,053 bp, a small single-copy region (SSC) of 12,635 bp, and a pair of inverted repeats (IRa and IRb) regions of 30,234 bp. The chloroplast genome with 37.95% GC content, contained 134 genes, including 90 protein-coding genes, 8 rRNA genes, and 36 tRNA genes. Phylogenetic analysis with the reported chloroplast sequences shows that *D. spathacea* is more closely related to *Spathodea campanulata.*

*Dolichandrone spathacea* (L. F.) K. Schum., a semi-mangrove plant belonging to the genus *Dolichandrone* of the Bignoniaceae, can grow in the waterlogged land of the beach and estuary as well as in land unaffected by tides (Lin 2001). It is an excellent coastal shelterbelt tree with certain ecological and economic value (Tian et al. 2011). There have been no reports on the complete chloroplast genome of *D. spathacea* since the publication of this species. Here, we assembled the complete chloroplast genome of *D. spathacea* and analyzed its phylogenetic relationship with other Bignoniaceae species. The chloroplast genome information can be further applied to conservation genetics, phylogeny, and breeding of this plant species.

Fresh young leaves of *D. spathacea* were collected from Qiao-Dangan Islands Provincial Mangrove Nature Reserve Management Agency, Zhuhai, Guangdong, China (N: 22°25′44.990″, E: 113°38′25.746″). Voucher specimen was deposited in the Herbarium of Beijing Forestry University (BJFU) (Ting Yu, yuting@bjfu.edu.cn) under collection numbers of *GWBDS001*. The chloroplast sequence was obtained through the high-throughput sequencing and splicing technology of genomic data (Yu et al. 2011). Genomic DNAs were extracted from fresh leaves using Plant Genomic DNA Kit (DP305). These DNAs were manufactured to average 150 bp paired-end (PE) library and sequenced on the Illumina Novaseq Sequencing platform. *De novo* assembly was used to reconstruct the chloroplast genomes using SPAdes version 3.6.1(Bankevich et al. 2012). *De novo* assembled chloroplast contigs were concatenated into larger contigs using Sequencher software (Gene Codes Inc., Ann Arbor, MI). Automatic annotation of the chloroplast genomes was generated by CpGAVAS2 (Shi et al. 2019). The draft annotations given by CpGAVAS2 were then manually corrected using the Artemis software and other plastid genomes for comparison (Rutherford et al. 2000).

The chloroplast genome of *D. spathacea* (Genbank accession no.MW173028) was a circular molecular genome with a size of 159,156 bp in length, 86,053 bp of a large single-copy (LSC) and 12,635 bp of small single-copy (SSC) regions were separated by 30,234 bp of inverted repeat (IR) regions. It contained 134 genes (90 protein-coding genes, 8 rRNA genes, and 36 tRNA genes). The overall GC content of the chloroplast genome was 37.95% and those in the LSC, SSC, and IR regions were 36.18%, 33.30%, and 41.43%, respectively.

In order to understand the phylogenetic relationship between *D. spathacea* and related taxa, the complete chloroplast of 15 species belonging to the Bignoniaceae family and two outgroups was used for phylogenetic analysis, they were downloaded from the NCBI GenBank database and aligned by MAFFT version 7.037 (Kazutaka Katoh, Japan) (Katoh et al. 2002). Phylogenetic relationship was inferred from maximum likelihood (ML) and Bayesian inference (BI) based on complete genome sequences. BI was performed with MrBayes version 3.2.7 (Ronquist et al. 2012) under the GTR + G + I model with 10,000 bootstrap and ML method was performed with IQ-TREE under the TVM + F + R4 model with 1000 bootstrap values in the PhyloSuite version 1.2.2 (Zhang et al. 2020). Two results gave the same topologies. The tree showed that *D. spathacea* was closely related to *Spathodea campanulata* (MN106255) which also belongs to the Paleotropical alliance (Figure 1) (Olmstead et al. 2009). In this article, we first reported the complete chloroplast genome of *D. spathacea*, which will provide fundamental genetic resources for studying this important species as well as resolving its phylogenetic evolution.

**Figure 1.**
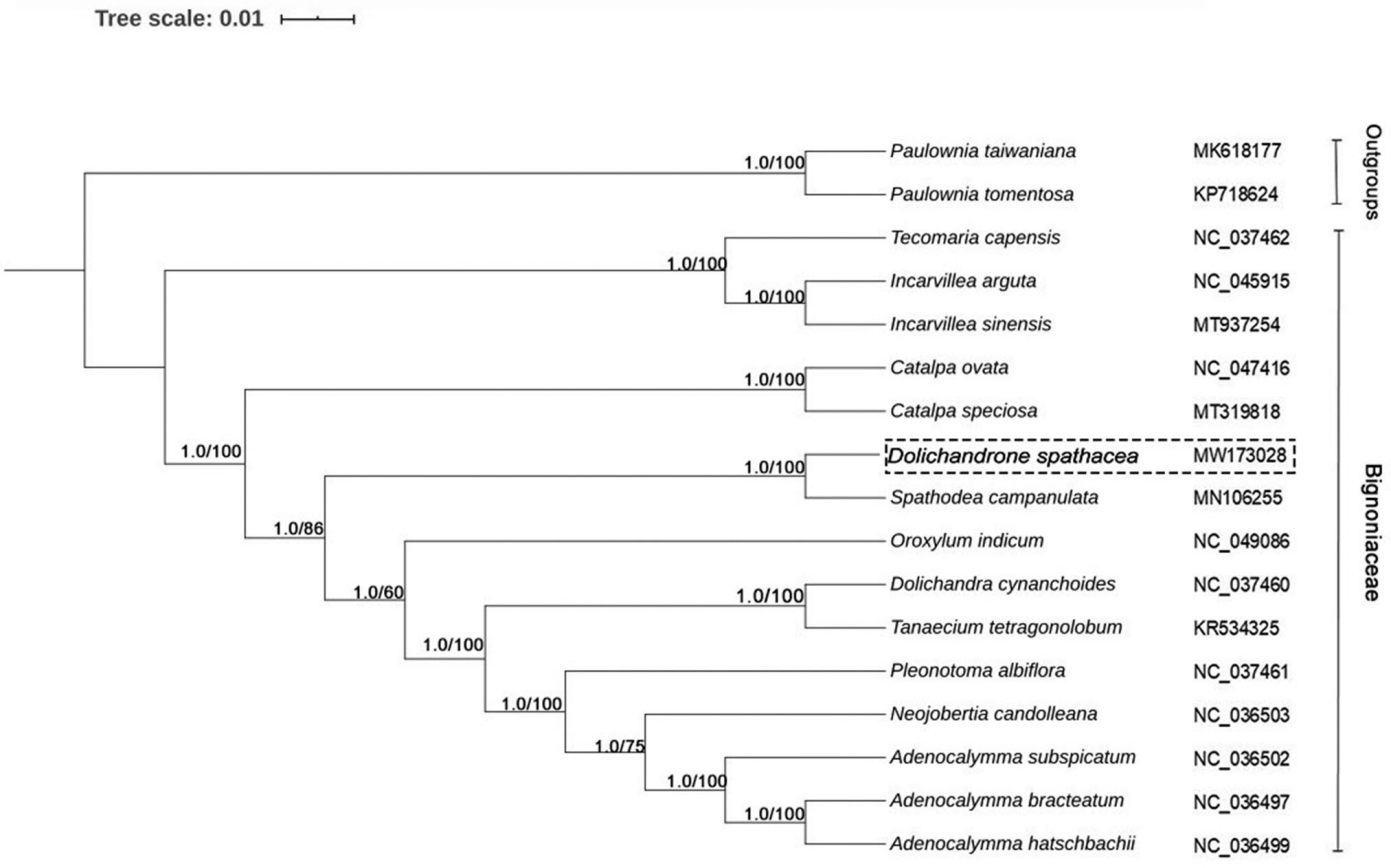
Phylogenetic relationship of the 17 species inferred from Bayesian inference (BI) and maximum likelihood (ML) based on complete genome sequences. PP values for Bayesian analysis and bootstrap values of ML analyses are shown at each node.

## Data Availability

The genome sequence data that support the findings of this study are openly available in GenBank of NCBI at https://www.ncbi.nlm.nih.gov/ under the accession no. MW173028. The associated BioProject, SRA, and Bio-Sample numbers are PRJNA703408, SRR13757104, and SAMN18016863, respectively.
